# Hyperacute Corticosteroid Treatment of Optic Neuritis at the Onset of Pain May Prevent Visual Loss: A Case Series

**DOI:** 10.1155/2011/815068

**Published:** 2011-06-30

**Authors:** G. T. Plant, N. A. Sibtain, D. Thomas

**Affiliations:** ^1^Department of Neuro-Ophthalmology, The National Hospital for Neurology and Neurosurgery, London WC1N 3BG, UK; ^2^Department of Neuro-Ophthalmology, Moorfields Eye Hospital, London EC1V 2PD, UK; ^3^The Medical Eye Unit, St Thomas' Hospital, London SE1 7EH, UK; ^4^Department of Neuro-Radiology, King's College Hospital, London SE5 9RS, UK

## Abstract

*Aim*. To show that high-dose corticosteroids may prevent visual loss in patients with optic neuritis (ON) treated at the prodromal, hyperacute, phase of retrobulbar pain. *Method*. Prospective case series: patients were recruited with a history of ON associated with pain. The patients were advised to report immediately to the investigators should the pain recur in either eye. Where possible, orbital magnetic resonance imaging (MRI) was performed to confirm a recurrence of ON and treatment with high-dose corticosteroids was commenced. Visual function and the patient's subjective account were monitored. *Results*. Eight patients (including cases of MS, CRION and NMO) presented in the hyperacute phase. MRI confirmed optic nerve inflammation in 5/5. Treatment was commenced immediately, and, in all cases, no visual loss ensued. *Conclusion*. MRI can be used to confirm acute optic neuritis prior to visual loss in the hyperacute phase. We suggest that treatment with high-dose corticosteroids may abort the attack and prevent loss of vision in patients with ON who are treated at the onset of pain. This has potential implications for the management of acute ON and also for our understanding of the pathogenesis and potential therapeutic targets in the neuroinflammatory conditions associated with ON.

## 1. Introduction

Subacute loss of vision accompanied by pain is most commonly due to optic neuritis (ON). Demyelinating optic neuritis—as occurs in association with multiple sclerosis (MS)—is the most common cause of acute and reversible visual failure in young adults of Northern European, North American, and Australasian origin and is second only to glaucoma as the most common acquired optic nerve disorder in persons younger than 50 years old. Optic neuritis is the initial presentation in 15% to 20% of cases of MS, and 38% to 50% of patients with MS develop optic neuritis at some point during the course of their disease [1]. Ten-year follow-up data from the Optic Neuritis Treatment Trial (ONTT) suggested that the prognosis for visual recovery is generally good; however, return of visual function is almost never complete [2]. The ONTT and other studies [3] have confirmed that the use of corticosteroids in the acute phase of optic neuritis shortens the time to recovery but has no effect on the final visual outcome. The results of these treatment trials have considerably altered the practice patterns of ophthalmologists and neurologists: in particular the use of corticosteroids in MS-associated optic neuritis has declined considerably [4, 5]. 

Optic neuritis also occurs in patients who have no evidence of MS. In some patients, the clinical phenotype is no different but others follow a very different clinical course. The term “chronic relapsing inflammatory optic neuropathy” (CRION) has been used to distinguish a type of optic neuritis characterised by pain and visual loss in which the symptoms recur when corticosteroids are withdrawn in the same or fellow eye; this does not occur in MS-associated optic neuritis. Kidd et al. [6] presented a series of 15 patients and suggested that the degree of visual loss in CRION is more severe than in demyelinating optic neuritis in general, usually with bilateral sequential involvement of both optic nerves. In this series, treatment with corticosteroids resulted in rapid resolution of pain and improvement in vision, with relapses when treatment was withdrawn or visual loss with abrupt steroid withdrawal. Long-term immunosuppression with agents such as azathioprine is often indicated. Some cases of isolated optic neuritis without evidence of MS may be cases of neuromyelitis optica (NMO) in whom spinal cord lesions do not occur, have not yet occurred, or have been prevented by long-term immunosuppression [7]. However, the proportion of such cases found to have the NMO-IgG antibody (anti-aquaporin-4) is low—around 5% only [8] as opposed to 70% in cases of NMO with both myelitis and optic neuritis. 

Optic neuritis differs from all other MS relapses, such as those affecting the spinal cord or brainstem, in that there is in 90% of cases a period of retrobulbar pain which may precede loss of visual function. Such pain is also common in non-MS optic neuritis including CRION and NMO. This therefore provides a unique opportunity to suppress the inflammatory lesion at an earlier phase in its evolution than in any of the published trials. The following cases suggest that treatment with a course of high-dose corticosteroids in patients who present with pain *before *the onset of visual loss may abort the pathological process and completely prevent the occurrence of any visual loss and, by inference, improve the final visual outcome.

## 2. Methods and Materials

Patients with decreased vision from a previous episode of optic neuritis were advised to present in what we are referring to as the “hyperacute” phase, that is, at the onset of retrobulbar pain before the onset of visual loss. When logistically possible, orbital magnetic resonance imaging was carried out using short-tau inversion recovery (STIR) or T2 weighted imaging and also T1-weighted Gadolinium-enhanced images with fat suppression to confirm a recurrence of optic neuritis. Treatment with a course of high dose corticosteroids was commenced using either an oral or an intravenous regimen dependent upon what was immediately available. This study is a case series and not a controlled clinical trial. Documentation of visual function was carried out as was practicable in a clinical setting, but we also relied upon the patient's subjective account of whether or not vision deteriorated following the institution of treatment. The cases reported are eight sequential cases managed in this manner: no case treated with corticosteroids in the hyperacute phase has lost vision.

## 3. Results

Eight patients with decreased vision from optic neuritis presented in the hyperacute phase with recurrent retrobulbar pain before visual loss. Where performed magnetic resonance imaging confirmed optic nerve enhancement. High-dose corticosteroids treatment was commenced immediately, and, in all cases no visual loss occurred. The clinical presentation and evolution of the visual symptoms in these eight patients is detailed in the case series and figures below. 

### 3.1. Case 1: Chronic Relapsing Inflammatory Optic Neuropathy

**Figure 1:**
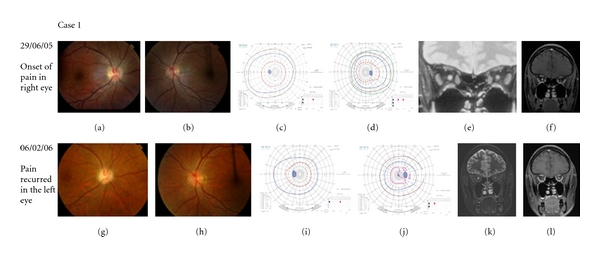
Disc photographs, Goldmann visual fields, and MR images of patient 1. (a)–(f) represent the initial episode in the right eye and (g)–(l) represent the recurrence in the left eye. Figure (e) is a coronal STIR image showing high signal within the right optic nerve, and Figure (f) is a coronal T1 fat saturation postgadolinium image showing associated optic nerve enhancement. (k) and (l) are coronal STIR and T1 postgadolinium images showing left optic nerve swelling and enhancement.

A 34-year-old female of Australasian ancestry, born in the UK, presented with subacute visual loss in the right eye preceded by retroocular pain. Vision deteriorated to no perception of light; vision in the unaffected (left) eye was 6/5 with normal perimetry. Magnetic resonance (MR) imaging performed 2 weeks after the onset of her symptoms showed hyperintensity on STIR images and Gadolinium enhancement of the intraorbital portion of the right optic nerve (Figures 1(e) and 1(f)). The brain appeared normal. A lumbar puncture was performed: the cerebrospinal fluid (CSF) analysis was normal apart from minimal elevation in the number of lymphocytes (5/mm^3^). On CSF protein electrophoresis, one oligoclonal band was found in both CSF and serum and one or two further bands were found in the CSF only. Serological tests for NMO were negative. The diagnosis was made of non-MS optic neuritis. 

The patient was treated with 1 g of Methyl Prednisolone intravenously for 3 days and with 60 mg of oral prednisolone for 3 weeks thereafter, at which point there had been good improvement in the right peripheral field. Her right visual acuity remained at 6/60 with a dense central scotoma, and the dose of prednisolone was steadily reduced as it was considered unlikely to improve further. Eventually, the daily dose was 7.5 mg and the signs were stable with no new symptoms. Repeat MR imaging was performed after one month at this dose and showed no persistent or recurrent enhancement. It was therefore considered reasonable to gradually reduce the corticosteroid dose further, that is, by 1 mg every two weeks. This proceeded uneventfully, and two months later the steroids were discontinued by which time the daily dose had been reduced to 1 mg. 

*One day *after stopping the steroid treatment, the patient noticed pain on moving the *left *eye but no loss of vision. The patient had been instructed to attend immediately in this eventuality and hence presented to Eye Casualty within 48 hours where she was noted to have no change in her visual acuity, normal colour vision, and normal Goldmann perimetry. MR imaging showed STIR hyperintensity and enhancement of the left optic nerve (Figures 1(k) and 1(l)). The patient was again treated with the same corticosteroid regimen. The pain resolved within a few hours, and there was no loss of vision, not subjectively, not on acuity and not on visual field examination. Her vision remains unchanged after 6 months follow-up and long-term immunosuppression with Azathioprine has been initiated. 

### 3.2. Case 2: Multiple-Sclerosis-Associated Optic Neuritis

A 46-year-old woman of Latin American ancestry noticed an acute onset of pain on moving her right eye followed by a decrease in her right eye vision 3 days later. 

Vision on the right deteriorated to hand movements but on the left was 6/5. By the time MR imaging was performed, a month had elapsed and her right visual acuity had improved to 6/18 but she was still only able to read 2/16 Ishihara colour plates and unable to see the I2e isopter on Goldmann visual field testing. Her left visual acuity was 6/5 and Goldmann field normal. MR imaging revealed multiple lesions typical of multiple sclerosis in the brain and cervical spinal cord. A diagnosis of MS-associated optic neuritis was made. 

**Figure 2:**
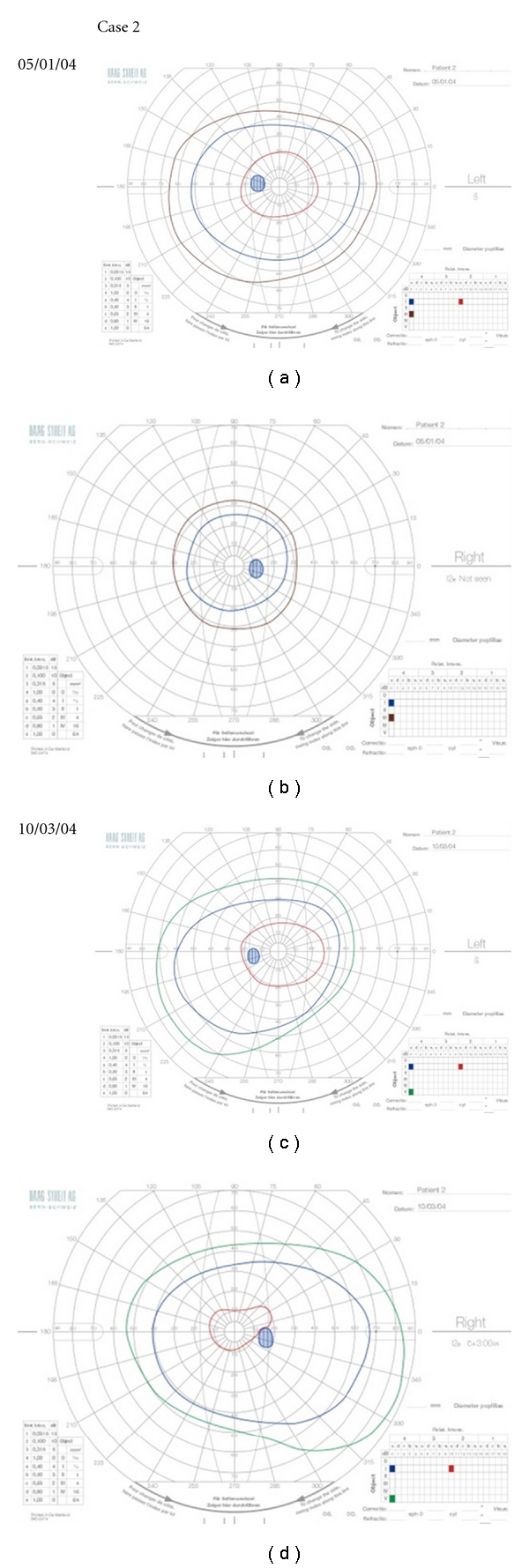
Goldmann visual fields performed on patient 2 at presentation of a recurrent episode of left retroorbital pain (a) and 2 months following treatment with intravenous methylprednisolone (c). The right eye had sustained an episode of optic neuritis previously (b and d).

The patient returned one month later with pain on moving her left eye for two days. Visual acuity remained 6/5, and Goldmann perimetry was normal (Figure 2(a)). The left optic disc appearance was also normal. The patient was treated immediately with intravenous Methylprednisolone 1 g daily for 3 days with no oral taper. The pain on eye movement, which she reported as being identical to that experienced previously disappeared immediately without there being any subjective deterioration in vision. The patient attended for review one month later by which time she had had an episode of sensory loss on her face. Her left visual function and visual field had remained unchanged on follow-up 2 months after treatment with intravenous methylprednisolone (Figure 2(c)).

### 3.3. Case 3: Chronic Relapsing Inflammatory Optic Neuropathy

**Figure 3:**
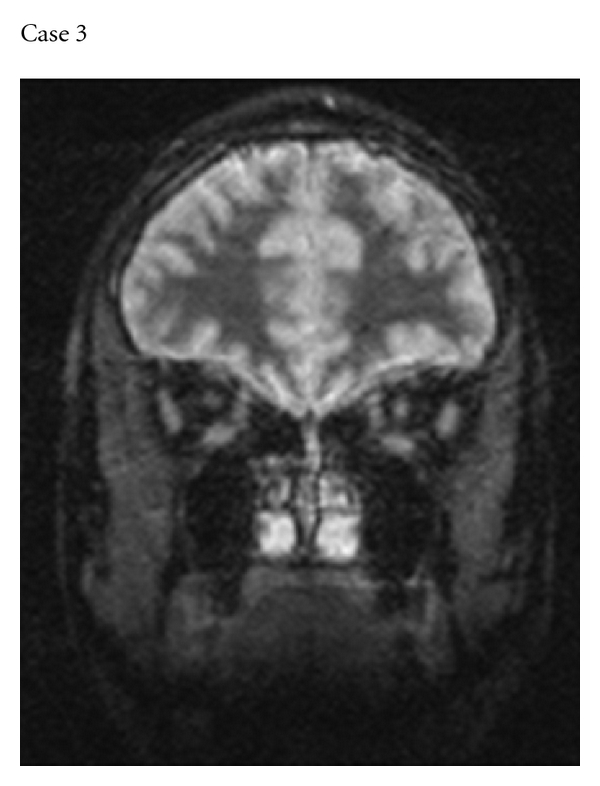
Coronal STIR image showing high signal within the optic nerve corresponding to the side of the pain in Case 3.

A 27-year-old female finance officer of African-Caribbean parentage, born in the UK, developed pain on moving the left eye. This progressed over a week and at its worst was severe enough to keep her awake at night. Two weeks from the onset of the pain, the vision in her left eye became blurred and progressed to no perception of light over 3 days. Over a month, there was modest spontaneous improvement in vision to hand movements only. The optic disc was swollen, and there was a dense left relative afferent pupillary defect. MR imaging revealed STIR hyperintensity within the left optic nerve; no abnormality was detected within the brain (Figure 3). Gadolinium enhancement was not undertaken. CSF was normal and negative for oligoclonal bands. A diagnosis of non-MS optic neuritis was made. She was treated with intravenous methylprednisolone 1 g daily for 3 days followed by oral prednisolone 60 mg daily. There was modest further improvement in her vision only. The dose of prednisolone was reduced gradually and discontinued entirely after 3 months. 

Two weeks later, the patient developed recurrent pain on moving her left eye, exactly as she had previously experienced. The pain continued for 3 days at which point she was seen in the clinic and treatment was restarted at a dose of 60 mg prednisolone daily. The vision did not deteriorate. A month later, she had discontinued prednisolone, and a few days later she developed pain on moving the as yet unaffected right eye. After a few days of pain, prednisolone was restarted and, once again, the pain resolved without loss of vision. No imaging was performed in these two episodes, and we are relying on the patient's subjective report of no deterioration in vision. The patient was intolerant of azathioprine and therefore commenced treatment with mycophenolate mofetil. The patient was maintained on this for 2 years but has since been lost to followup. NMO-IgG status is not known. 

### 3.4. Case 4: Chronic Relapsing Inflammatory Optic Neuropathy in Neuromyelitis Optica

A 34-year-old African-Caribbean man, born in the UK, presented with a 2-week history of left retroorbital pain and deterioration in vision. The right visual acuity was poor due to myopia and amblyopia: a right divergent strabismus had been present since childhood. On admission, his visual acuity was perception of light only on the left and finger counting on the right. The appearance of the left fundus was normal. On the right, there was a staphyloma. MR imaging revealed no abnormality in the brain but there was enhancement of the left optic nerve, both its intracranial and intraorbital portions. The CSF was negative for oligoclonal bands. NMO-IgG was positive. 

The patient was treated with intravenous methyl prednisolone followed by prolonged treatment with oral prednisolone. Two weeks later, Goldmann perimetry revealed a dense central scotoma, but after a further 2 weeks, the scotoma was no longer detectable. The pain resolved and visual acuity improved to 6/9 with 12/17 Ishihara plates read correctly. The dose of prednisolone was gradually reduced. 

**Figure 4:**
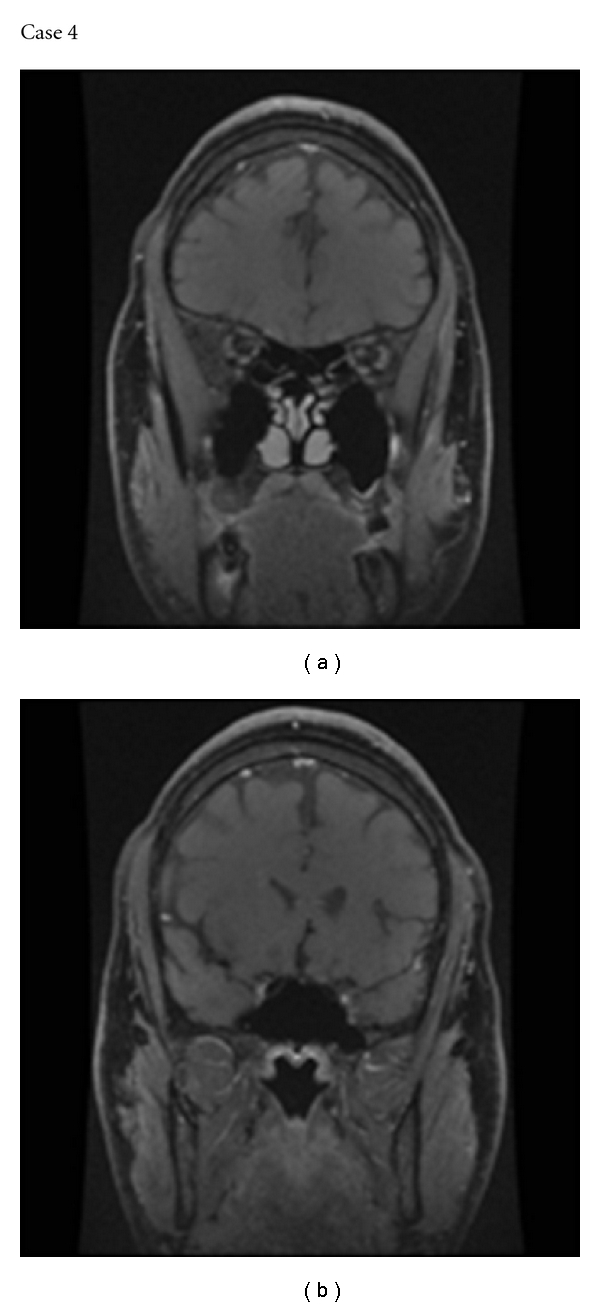
MR imaging of Case 4 showing extensive enhancement of the left optic nerve both intraorbitally (a) and intracranially (b).

He presented again several months later. At that point the patient was taking 20 mg of prednisolone once daily. The left retroorbital pain had recurred, but there was no subjective deterioration in vision. In fact, the visual acuity was 6/7.5, N4.5 with one error on the Ishihara plates and the Goldmann field was only mildly, generally depressed. MR imaging showed extensive enhancement of the left optic nerve both intraorbitally and intracranially (Figure 4). The patient was treated with intravenous methylprednisolone. His pain resolved, and there was no change in his vision. 

He was most recently seen a year later. His vision in the left eye remained good, and he was on reducing doses of prednisolone. 

### 3.5. Case 5: Multiple-Sclerosis-Associated Optic Neuritis

A 33-year-old white female patient presented with right optic neuritis with pain. No treatment was given, she had a poor outcome with a visual acuity of 1/60. The following year, she had a spinal cord relapse and MR imaging of the brain revealed appearances typical of multiple sclerosis. A year later, she developed similar pain on moving the right eye again and presented that day for advice. There had been no change in her vision. The patient was treated with oral methylprednisolone 500 mg daily for 5 days. Over 3 days, the pain resolved. There was no subjective deterioration in her vision. 

### 3.6. Case 6: Recurrent Isolated Optic Neuritis

**Figure 5:**
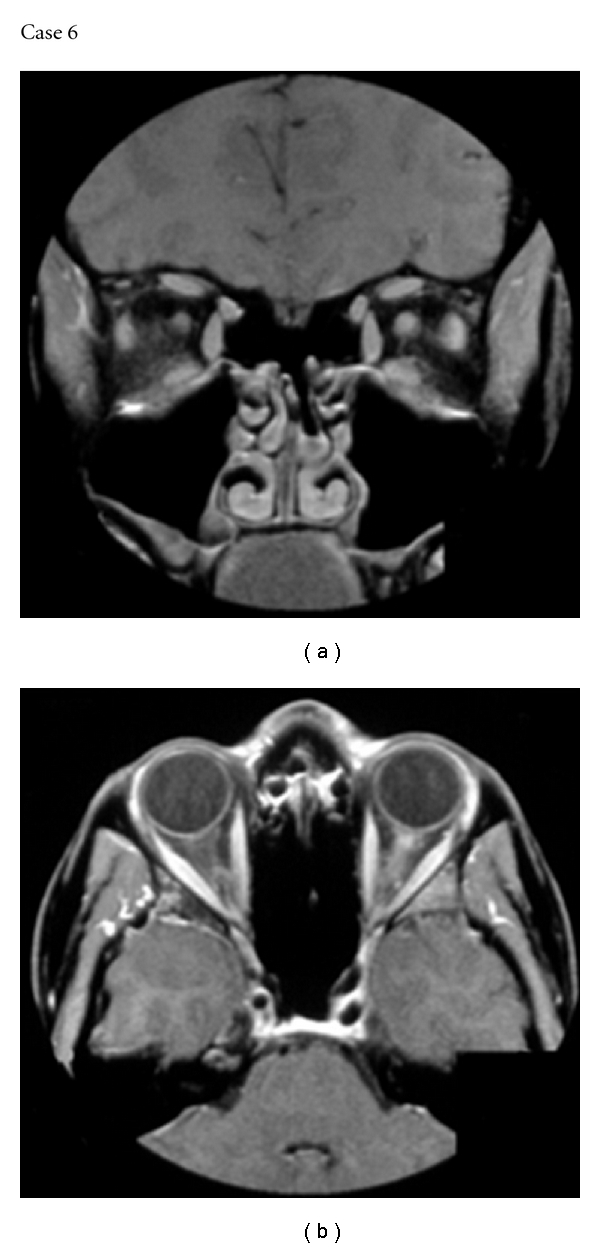
MR images of Case 6 showing gadolinium enhancement of the left optic nerve 24 hours after the onset of left retroorbital pain.

A 33-year-old female presented with a typical episode of right optic neuritis with pain and good recovery of vision. Three months later, she presented with a 24-hour history of pain on moving the left eye. MR imaging of the orbits with Gadolinium enhancement confirmed acute optic neuritis (Figure 5). She was started immediately on oral prednisolone 40 mg daily, and, within 24 hours, the pain had resolved and no loss of vision occurred. The patient remained on a tapering dose of prednisolone for 3 weeks. MR imaging of the brain and spinal cord revealed no abnormalities to indicate multiple sclerosis. Further episodes of optic neuritis have occurred over 6 years of follow-up and she has had sensory symptoms in her limbs. Repeat MR imaging and CSF examination has not confirmed a diagnosis of multiple sclerosis, and all other routine blood tests including NMO IgG testing have proven normal. The patient therefore has a diagnosis of isolated recurrent optic neuritis (RION) of unknown cause. 

### 3.7. Case 7: Chronic Relapsing Inflammatory Optic Neuropathy

A 32-year-old Moroccan lady developed symptoms of left optic neuritis, consisting of pain on eye movement and progressive visual loss. The patient was given no treatment and her vision deteriorated to no perception of light. The following year, she developed similar symptoms on the right whilst in Morocco but on this occasion she was treated with corticosteroids. Her vision returned to normal but after 2 weeks the steroids were discontinued and, her vision deteriorated again. On coming under our care her vision was counting fingers and further treatment with corticosteroids produced an improvement in vision to 6/12 with inferior field loss and 11/13 Ishihara plates seen. The patient was maintained on immunosuppression but as the dose of corticosteroids was reduced her vision deteriorated again and she was treated with a conventional course of intravenous cyclophosphamide. MR imaging showed enhancement of the right intraorbital optic nerve. There were no brain or spinal cord lesions. Oligoclonal bands were found in both CSF and serum but with fewer bands in the serum. NMO antibody was negative. She was eventually maintained on 10 mg of prednisolone daily. Two years later, she presented again with pain on movement of her right eye. There had been no subjective deterioration in vision. Urgent MR imaging of the orbits showed enhancement of the right optic nerve. The patient was given 3 days of intravenous methylprednisolone, and the dose of oral prednisolone continued at 60 mg daily. No deterioration in her vision occurred subjectively, and this was confirmed by serial measurements of visual acuity and the Ishihara test. 

### 3.8. Case 8: Neuromyelitis Optica (NMO-IgG Negative)

A 23-year-old white female was admitted with a two-week history of pain on eye movement followed by visual loss in the left eye and a one-week history of similar symptoms in the right eye. On admission, her vision was counting fingers in the right eye and no perception of light in the left. The patient also gave a one-day history of sensory symptoms in her legs and saddle area with urinary retention. There was a sensory level to pinprick at T8. The appearance of the optic disc was normal. MR imaging showed T2 hyperintensity and enhancement of both optic nerves intracranially just anterior to the chiasm. The brain appeared normal. MR imaging of the spinal cord showed a T2 hyperintense lesion within the distal spinal cord extending across 3 to 4 vertebral segments. Cerebrospinal fluid examination revealed a white cell count of 59/mm^3^, 78% of which were lymphocytes and the remainder polymorphs. Matched oligoclonal bands were present in CSF and serum. 

**Figure 6:**
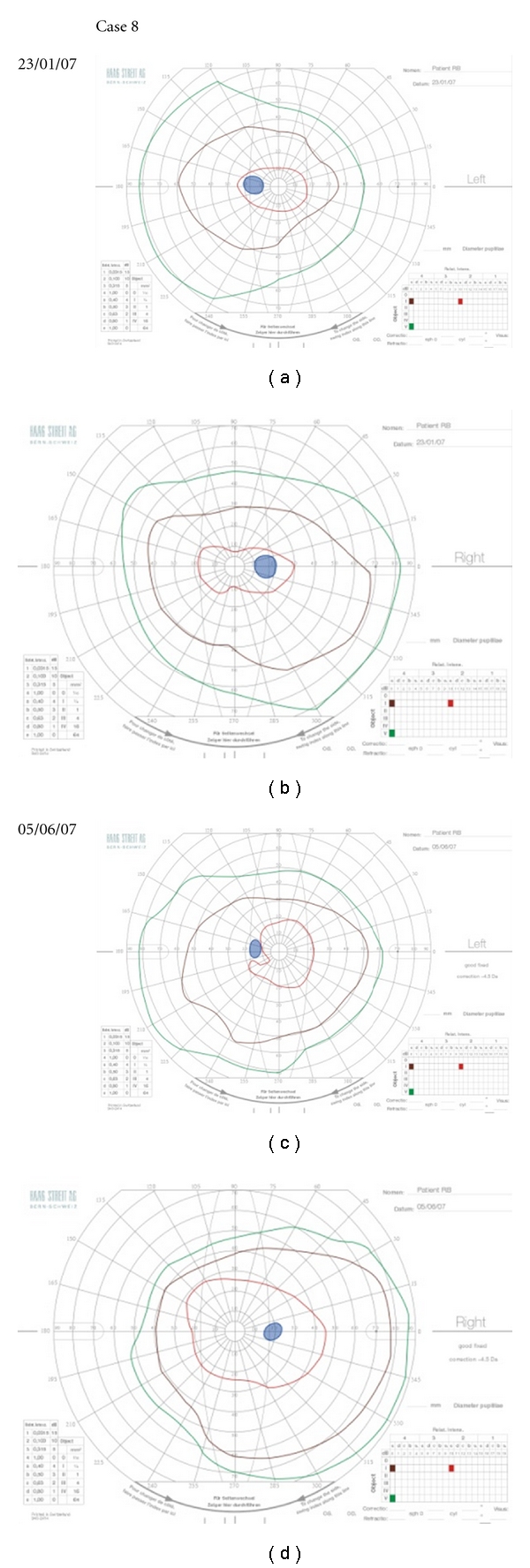
Goldmann visual fields showing minimal change in the left eye 1 month after a recurrent episode of optic nerve swelling (c) compared with visual fields performed 4 months before this recurrence (a). The contemporaneous right visual fields (b and d) are presented for comparison.

The patient was treated with high-dose methylprednisolone followed by oral prednisolone and made an excellent visual recovery, and when seen three months later, her visual acuity was 6/6 in the right eye and 6/9 in the left, N5 bilaterally with no mistakes made on the Ishihara plates with either eye. There was mild bilateral temporal pallor of the optic discs. Oral prednisolone was discontinued after six-month treatment, and she remained entirely well until a year later when she developed pain on moving the left eye and MR imaging of the orbits showed enhancement of the left optic nerve/sheath complex. No loss of vision occurred, and the optic disc appearance was unchanged. She was treated immediately following the scan with high-dose intravenous methylprednisolone followed by oral prednisolone. At the time steroids were given, the visual acuities were recorded as 6/5, N5 bilaterally with full colour vision; there was no subjective deterioration in vision. Goldmann visual fields 4 months before and 1 month after this episode are shown in Figure 6. A mild spinal cord relapse occurred a week later. She subsequently discontinued all therapy and remains well. NMO antibody testing has been negative.

## 4. Discussion

Pain in the distribution of the first division of the trigeminal nerve and pain on eye movement are often reported by patients with acute optic neuritis [9, 10]. In the Optic Neuritis Treatment Trial (ONTT) for example, pain on eye movements was reported by 92% of participants, 87% of whom noted a worsening of the pain with eye movement [11]. However, the pathophysiological basis for this phenomenon has not been determined. Conditions such as papilloedema cause extensive optic nerve swelling and sheath enlargement but are not associated with periocular pain, suggesting that simple distension of the meninges is unlikely to be a primary source of pain in optic neuritis [12]. 

Lepore [13] suggested that pain on eye movement in optic neuritis occurs because of the close association of the optic nerve sheath and the sheaths of the superior and inferior recti at the orbital apex. These authors hypothesised that a mechanical irritation of the inflamed optic nerve sheath is caused by traction when the extraocular muscles contract resulting in pain on eye movement. 

Fazzone et al. [14] subsequently used MR imaging in patients with acute optic neuritis and found that the incidence of pain may be dependent on the localisation of the inflammation. In this study, periorbital pain and pain on eye movement were found to be more frequent when enhancement of the orbital or retrobulbar segments of the nerve was seen and pain was twenty times more likely to be absent when there was no enhancement of the orbital segment. 

Several trials have shown that treatment of acute optic neuritis with high-dose, intravenous corticosteroids followed by oral corticosteroids accelerated visual recovery but provided no long-term benefit to vision [2, 3]. A meta-analysis of trials evaluating methylprednisolone with total dose greater than 3000 mg administered intravenously supported this view [15]. It was found that the relative risk of normal visual acuity with intravenous corticosteroids compared with placebo was 1.06 (95% CI 0.89 to 1.27) at six months and 1.06 (95% CI 0.92 to 1.22) at one year. The authors concluded that there was no conclusive evidence of benefit in terms of recovery to normal visual acuity, visual field, or contrast sensitivity with either intravenous or oral corticosteroids at the doses evaluated in trials included in the paper. However, these studies have concentrated exclusively on MS-associated optic neuritis and the situation is very different in NMO and cases of optic neuritis of unknown aetiology where corticosteroids may indeed improve the outcome. 

The eight cases presented above show no subjective loss of vision and, when measurements have been practicable, there has been no change in visual acuity, in colour vision nor in visual field following treatment with high-dose corticosteroids despite clear imaging evidence in 5/8 cases of optic nerve inflammation.

## 5. Conclusions

We are proposing that patients who have had optic neuritis, whether in the context of MS or otherwise, may benefit from hyperacute treatment with corticosteroids given at the phase of retrobulbar pain in subsequent episodes. Our experience in the cases reported here is that, when treatment is given in this way, patients may not lose vision to any significant degree, perhaps not at all, thus clearly predicting a more favourable outcome than if steroids are given later or not given.

There are many potential causes of ocular pain or discomfort but if patients have experienced optic neuritis previously, they will be in a position to tell us whether the pain has the same qualities. Furthermore, MR imaging can be used to confirm acute optic neuritis as in the five cases described above in whom scans were performed. We propose a new trial with a novel protocol looking at corticosteroid treatment in the hyperacute phase preceding the onset of visual loss. It is unlikely that pain preceding optic neuritis could ever be recognised in a first, clinically isolated attack but, if patients are appropriately instructed, presentation during this phase in subsequent attacks would be feasible.

## References

[1] Gilbert ME, Sergott RC (2007). New directions in optic neuritis and multiple sclerosis. *Current Neurology and Neuroscience Reports*.

[2] Beck RW, Gal RL, Bhatti MT (2004). Visual function more than 10 years after optic neuritis: experience of the optic neuritis treatment trial. *American Journal of Ophthalmology*.

[3] Kapoor R, Miller DH, Jones SJ (1998). Effects of intravenous methylprednisolone on outcome in MRI-based prognostic subgroups in acute optic neuritis. *Neurology*.

[4] Trobe JD, Sieving PC, Guire KE, Mark Fendrick A (1999). The impact of the optic neuritis treatment trial on the practices of ophthalmologists and neurologists. *Ophthalmology*.

[5] Katz B, Trobe JD, Beck RW (1995). The optic neuritis treatment trial: implications for clinicians. *Seminars in Ophthalmology*.

[6] Kidd D, Burton B, Plant GT, Graham EM (2003). Chronic relapsing inflammatory optic neuropathy (CRION). *Brain*.

[7] Lennon PVA, Wingerchuk DM, Kryzer TJ (2004). A serum autoantibody marker of neuromyelitis optica: distinction from multiple sclerosis. *Lancet*.

[8] Petzold A, Pittock S, Lennon V, Maggiore C, Weinshenker BG, Plant GT (2010). NMO-IgG (aquaporin-4) autoantibodies in immune mediated optic neuritis. *Journal of Neurology, Neurosurgery and Psychiatry*.

[9] Agostoni E, Frigerio R, Protti A (2005). Controversies in optic neuritis pain diagnosis. *Neurological Sciences*.

[10] Protti A, Spreafico C, Frigerio R (2004). Optic neuritis: diagnostic criteria application in clinical practice. *Neurological Sciences*.

[11] Beck RW (1991). The clinical profile of optic neuritis: experience of the optic neuritis treatment trial. *Archives of Ophthalmology*.

[12] Hickman SJ, Toosy AT, Jones SJ (2004). A serial MRI study following optic nerve mean area in acute optic neuritis. *Brain*.

[13] Lepore FE (1991). Determinants of Pain in 101 Eyes With Optic Neuritis. *Archives of Neurology*.

[14] Fazzone HE, Lefton DR, Kupersmith MJ (2003). Optic neuritis: correlation of pain and magnetic resonance imaging. *Ophthalmology*.

[15] Vedula SS, Brodney-Folse S, Gal RL, Beck R (2007). Corticosteroids for treating optic neuritis. *Cochrane Database of Systematic Reviews*.

